# Effect of GP visits in the compliance of preventive services: a cross-sectional study in Europe

**DOI:** 10.1186/s12875-024-02400-w

**Published:** 2024-05-15

**Authors:** Sara Ares-Blanco, Juan A. López-Rodríguez, Elena Polentinos-Castro, Isabel del Cura-González

**Affiliations:** 1 Federica Montseny Health Centre, Gerencia Asistencial Atención Primaria, Servicio Madrileño de Salud, Madrid, Spain; 2 Medical Specialties and Public Health, School of Health Sciences, Rey Juan Carlos University, Alcorcón, Madrid, Spain; 3 Instituto de Investigación Sanitaria Gregorio Marañón, Madrid, Spain; 4 General Ricardos Health Centre, Gerencia Asistencial Atención Primaria, Servicio Madrileño de Salud, Madrid, Spain; 5 Primary Care Research Unit, Gerencia de Atención Primaria, Servicio Madrileño de Salud, Madrid, Spain; 6 Chronicity, Primary Care, and Health Promotion Networks (RICAPPS), ISCIII, Madrid, Spain; 7 Medical Specialties and Public Health Department, School of Health Sciences, Rey Juan Carlos, University, Alcorcón, Madrid, Spain; 8 Aging Research Center, Karolinksa Instituted, Stockholm, Sweden

**Keywords:** General Practice, Diagnostic Screening Programs, Preventive Health Services, Health Care disparities, Europe

## Abstract

**Background:**

Performing cardiovascular and cancer screenings in target populations can reduce mortality. Visiting a General Practitioner (GP) once a year is related to an increased likelihood of preventive care. The aim of this study was to analyse the influence of visiting a GP in the last year on the delivery of preventive services based on sex and household income.

**Methods:**

Cross-sectional study using data collected from the European Health Interview Survey 2013–2015 of individuals aged 40–74 years from 29 European countries. The variables included: sociodemographic factors (age, sex, and household income (HHI) quintiles [HHI 1: lowest income, HHI 5: more affluent]), lifestyle factors, comorbidities, and preventive care services (cardiometabolic, influenza vaccination, and cancer screening). Descriptive statistics, bivariate analyses and multilevel models (level 1: citizen, level 2: country) were performed.

**Results:**

242,212 subjects were included, 53.7% were female. The proportion of subjects who received any cardiometabolic screening (92.4%) was greater than cancer screening (colorectal cancer: 44.1%, gynaecologic cancer: 40.0%) and influenza vaccination. Individuals who visited a GP in the last year were more prone to receive preventive care services (cardiometabolic screening: adjusted OR (aOR): 7.78, 95% CI: 7.43–8.15; colorectal screening aOR: 1.87, 95% CI: 1.80–1.95; mammography aOR: 1.76, 95% CI: 1.69–1.83 and Pap smear test: aOR: 1.89, 95% CI:1.85–1.94). Among those who visited a GP in the last year, the highest ratios of cardiometabolic screening and cancer screening benefited those who were more affluent. Women underwent more blood pressure measurements than men regardless of the HHI. Men were more likely to undergo influenza vaccination than women regardless of the HHI. The highest differences between countries were observed for influenza vaccination, with a median odds ratio (MOR) of 6.36 (under 65 years with comorbidities) and 4.30 (over 65 years with comorbidities), followed by colorectal cancer screening with an MOR of 2.26.

**Conclusions:**

Greater adherence to preventive services was linked to individuals who had visited a GP at least once in the past year. Disparities were evident among those with lower household incomes who visited a GP. The most significant variability among countries was observed in influenza vaccination and colorectal cancer screening.

**Supplementary Information:**

The online version contains supplementary material available at 10.1186/s12875-024-02400-w.

## Background

Life expectancy at birth in the European Union (EU) was estimated at 81 years in 2018. As life expectancy increases, the burden of noncommunicable diseases (NCDs) grows. In 2017, the main causes of death in the EU included cardiovascular diseases and cancer [1]. The top risk factors for NCDs are unhealthy diet, tobacco use, alcohol abuse and physical inactivity. Health promotion and preventive care services aim not only to delay the onset of chronic diseases but also to achieve early detection to prevent complications. Preventive care accounted for 2.8% of health expenditures in the EU [2]. Influenza vaccination, blood pressure measurement and cancer screening have been shown to be cost-effective [3]. The World Health Organization (WHO) European region has developed an action plan to prevent and control NCDs in the region [4]. The plan includes promoting vaccination to prevent the exacerbation of NCDs; early detection of cervical, breast and colorectal cancer, and cardiometabolic risk assessment when presenting in primary care among other actions. In Europe, colorectal, cervical and breast screening have reduced the risk of mortality [5–7]. Other studies suggested that counselling to improve diet and physical activity behaviour brought modest benefits in cardiometabolic health outcomes in the general population [8].

Primary care serves as the first point of contact with the health system and typically reaches the whole population. An increased utilization of primary care services by patients with lower socioeconomic status has been described [9]. Preventive health services are provided mainly by primary care given that primary care is in a privileged position to ensure access to preventive care and address inequalities. General practitioners (GPs) usually perform opportunistic screening when patients consult for any reason, and the frequency of primary care visits not only improves participation in preventive care services but also reduces disparities [10]. Having at least one primary care visit a year is related to an increased likelihood of vaccination, mammography and colonoscopy [11].

The organization of health care systems in the EU influences the population’s access to preventive care services. There may be differences in coverage, financing systems, remuneration of service providers, access of providers to health care markets and access of patients to service providers in the EU [12, 13]. Primary care-oriented health services are associated with lower costs of care, improved access to more appropriate services, and reduced inequities in the population’s health [14–16]. Primary care is characterized by continuity of care, coordinated care and good communication between patients and doctors, which are related to better compliance with preventive care services, including immunization [17]. The aim of this study was to analyse the influence of visiting a GP in the last year on the delivery of preventive services (cardiometabolic screening, cancer screening and influenza vaccination) based on sex and household income in European countries.

## Methods

### Study design and population

This was a cross-sectional study using data collected as part of the survey conducted by the European Commission with the support of Eurostat (European Health Interview Survey: EHIS wave 2). It included 29 European countries (n: 242,212). The survey included a national representative population probability sample from each participant country between 2013 and 2015. Questionnaires were administered in face-to-face, by telephone or self-administered and sent by mail or internet. The details of the methodology are available on the Eurostat website [18, 19]. The detailed description of each variable, the handling of missing data by Eurostat, and the type of response possible for each question can be consulted in the survey manual, which has been published [18].

In this study, the selected population included adults between 25 and 75 years old given that some preventive services (cancer screenings) did not focus on very elderly patients as the target population. Participants belonged to EU members plus Iceland, Norway and the United Kingdom (the list of countries is available in Supplementary file 1). This study is reported as per the Strengthening the Reporting of Observational Routinely collected health Data (RECORD) Statement (see Supplementary file 2 RECORD Checklist).

### Variables

The explanatory variable, namely, GP visits, was measured with the following question: “When was the last time you consulted a GP on your own behalf?”. Face-to-face or remote assessments were considered. The number of visits to the GP was initially measured as a discrete variable and recoded as a dichotomous variable based on whether the patient had visited a GP during the last 12 months or not.

Covariates included the patient’s sex and household income (HHI). HHI was calculated from the total income of a household after tax deduction and household size. Respondents were assigned to terciles: low income, middle income or high income. Other variables were age, country, and urban area. Lifestyle factors were recorded using the WHO criteria as follows: active lifestyle (exercise practice of ≥ 2.5 h/week) [20], healthy diet (eating vegetables and fruits at least 4–6 days a week) [21], tobacco consumption (daily smoking) [22] and alcohol consumption (daily consumption over 20 g in women and 40 mg in men) [23]. Obesity (body mass index: BMI ≥ 30 kg/m^2^) was computed from anthropometric height and weight measurements [24]. Pre-existing diseases (hypertension, diabetes, ischaemic heart disease, stroke and renal disease) were self-reported. Self-perceived health (SPH) was reported by the individuals on a three-step scale: very good and good, fair, bad and very bad.

The use of preventive health care services, including cardiometabolic screening, flu vaccination, and cancer screening, served as the outcome variable. Cardiometabolic prevention was reported based on whether patients measured their glucose, cholesterol or blood pressure (BP) at least once in the past 5 years. Blood pressure measurement was included if it was measured by a health professional. If patients had chronic conditions (hypertension, diabetes, ischaemic heart disease, stroke and renal disease), the optimal glucose, cholesterol and BP screening was recorded as the number of measurements per year. The target population for cardiometabolic screening comprised 176,890 individuals for glucose measurement, 177,642 for cholesterol assessment, and 178,427 for blood pressure evaluation. The preventive care services of the different guidelines are provided in Supplementary file 3. Influenza vaccination was recorded as any shot in the last year in patients over 65 years and annually in patients with comorbidities (n: 100,834) [25]. Patients were considered to participate in colorectal cancer screening if faecal occult blood testing (FOBT) was performed once every 2 years from 50 to 74 years of age. FOBT could be replaced by colonoscopy. In this study, colonoscopy was considered any test performed throughout life with the same target populations as FOBT. The intended demographic for colorectal cancer were n: 126,276 individuals. Patients were considered to undergo mammography if any test was performed between 50 and 69 years of age with a biennial frequency (n: 106,260). The cervical smear test considered any test performed every 3 years from 25 to 64 years (n: 196,976).

### Analysis

Subject characteristics were described using descriptive statistics (means, standard deviations, medians, interquartile ranges and proportions). Missing data were excluded of the analysis as we performed a complete-case analysis. The target population for each prevention use indicator differed given the different target ages of each recommendation and indication. We examined associations across sexes and HHI in those who visited a GP in the last year using the χ² test.

We analyzed the impact of GP visits for each of the screenings using a multilevel logistic model (level 1: citizen, level 2: country), which includes adjustment for the sociodemographic (sex, age, household income) and clinical characteristics (healthy diet, use of tobacco, obesity, diabetes, hypertension, coronary heart disease, stroke, renal disease and self-perceived health) of the subjects, with the country as a random effect. The dependent variable was the cardiometabolic and cancer screenings, and the independent variable was GP visit in the last year. The random effect was quantified via the median OR (MOR) between countries [26], interpreted as the expected change (in medians) in cardiometabolic and cancer screening for a citizen who switches from one country to another country with increased risk. The intraclass correlation coefficient (ICC) was calculated as ICC = V_A_/(V_A_+V_I_). MOR was calculated following formula: MOR= [$\surd$ (2xV_A_) x 0.6745] ≈ exp (0.95 $\surd$V_A_). All tests were conducted at a significance level of 0.05. Statistical analysis was performed using STATA 16.0.

## Results

A total of 242,212 individuals were analysed. Population characteristics are summarized in Table 1 based on patients who attended their GP in the last year and those who did not. Supplementary file 4 lists the general characteristics according to sex and GP visit. Women represented 53.7% of the study population. Most of the subjects were aged 40–64 years (55.4%). Regarding household income, 43.9% of the sample reported a high income. Hypertension (22.4%) and obesity (20.7%) were the most common comorbidities. A total of 25.7% of the sample smoked, and 63.6% had a healthy diet.

**Table 1:** Population characteristics of participants who did or did not visit their GP in the last year

	All	GP visit in the last year	No GP visits in the last year	*P* value
**n**	242,212	175,047 (72.2)	67,165 (27.7)	< 0.001
Women*	129,967 (53.7)	99,257 (56.7)	30,710 (45.7)	
Men*	112,245 (46.3)	75,790 (43.3)	36,455 (54.3)	
Urban*	158,486 (65.5)	114,622 (65.6)	43,194 (65.5)	0.64
Age* 25–39 years	62,894 (26.0)	40,438 (23.1)	22,456 (33.4)	< 0.001
40–64 years	134,082 (55.4)	96,970 (55.4)	37,112 (55.3)	
65–74 years	45,236 (18.7)	37,639 (21.5)	7597 (11.3)	
Household income*				
Low	81,828 (36.1)	59,390 (36.3)	22,438 (35.4)	< 0.001
Middle	45,343 (20.0)	33,251 (20.3)	12,092 (19.1)	
High	99,656 (43.9)	70,886 (43.3)	28,770 (45.5)	
Self-perceived health*				
Very good and good	162,664 (69.1)	107,627 (63.0)	55,037 (85.0)	< 0.001
Fair	55,515 (23.6)	47,169 (27.6)	8346 (12.9)	
Bad and very bad	17,395 (7.4)	16,000 (9.4)	1395 (2.2)	
Lifestyle Factors*				
Healthy diet	154,047 (63.6)	113,912 (65.1)	40,135 (59.8)	< 0.001
Exercise	237,127 (97.9)	171,262 (97.8)	65,865 (98.1)	0.001
Tobacco	61,341 (25.7)	41,130 (23.8)	20,211 (30.7)	< 0.001
Alcohol abuse	6405 (2.8)	4149 (2.5)	2256 (3.6)	< 0.001
Chronic Conditions*				
Obesity	50,157 (20.7)	39,085 (22.3)	11,072 (16.5)	< 0.001
Diabetes	15,927 (6.7)	14,570 (8.4)	1357 (2.0)	< 0.001
Hypertension	53,725 (22.4)	47,780 (27.6)	5945 (9.0)	< 0.001
Coronary heart disease	9690 (4.1)	8889 (5.2)	801 (1.2)	< 0.001
Stroke	2710 (1.1)	2468 (1.4)	242 (0.4)	< 0.001
Renal disease	6182 (2.6)	5553 (3.2)	629 (1.0)	< 0.001
**Preventive Services**				
Cardiometabolic screening*	165,237 (92.4)	129,786 (96.0)	35,451 (81.2)	< 0.001
Glucose measurement*	139,863 (79.1)	111,553 (83.3)	28,055 (65.9)	< 0.001
Cholesterol measurement*	143,286 (80.7)	114,255 (85.0)	28,730 (67.2)	< 0.001
BP measurement*	161,193 (90.3)	127,078 (94.2)	33,770 (78.5)	< 0.001
Influenza vaccination under 65 yo with comorbidities *	7522 (19.2)	6878 (19.9)	644 (14.0)	< 0.001
Influenza vaccination in individuals over 65 yo*	18,138 (40.1)	15,817 (42.1)	2321 (30.6)	< 0.001
Colorectal cancer screening*	55,723 (44.1)	47,613 (48.2)	7665 (28.6)	< 0.001
Mammography*	36,417 (34.3)	30,505 (37.2)	5797 (24.3)	< 0.001
Cervical smear test*	75,088 (38.1)	58,482 (42.6)	16,435 (28.0)	< 0.001

*: n (%). GP: General Practitioner yo: years old

Note: The association between visiting a GP in the last year and cardiometabolic screening, cancer screening and influenza vaccination was assessed using the χ² test

Patients who visited a GP in the last year had more chronic conditions than those who did not. More women visited a GP in the last year compared to men (56.7% vs. 43.3%). Patients who did not attend their GP in the last year smoked more (30.7% vs. 23.8%) and more heavily consumed alcohol (3.6% vs. 2.5%) than those who did attend. Patients who visited a GP in the last year were more likely to have very good and good self-perceived health (85.0% vs. 65.1%).

Cardiometabolic screening was the most commonly conducted screening, with blood pressure measurement at 90.3%, cholesterol measurement at 80.7%, and glucose measurement at 79.1%. This was followed by colorectal cancer screening at 44.1%.

### GP visits and preventive care services based on sex and household income

Table 2 shows preventive care services based on attending GP practice, sex and income group. Individuals who visited their GP in the last year exhibited higher compliance with all preventive services than those who did not visit the GP. Individual characteristics of participants receiving preventive care services are provided in Supplementary file 5.

**Table 2:** Preventive services based on GP visit, sex and household income

			Glucose measurement† Target population: 176,890		Cholesterol measurement† Target population: 177,642	BP measurement†* Target population: 178,427	Influenza vaccination under 65 yo with comorbidities # Target population: *39,185*	Influenza vaccination in individuals over 65 yo # Target population: 45,209	Colorectal cancer screening ¶ Target population: 126,276	Mammography ‡ Target population: 106,260		Cervical smear test ## Target population: 196,976
**GP visit**	**HHI in** **Women**	Low (n:35,825)		22,628 (81.8)	23,141 (83.4)	26,219 (94.1)	1463 (19.3)	3593 (40.6)	9498 (46.1)	9922 (61.2)		18,651 (69.2)
		Middle (n:18,968)		12,062 (83.1)	12,347 (84.7)	13,877 (94.8)	627 (18.8)	1861 (40.8)	5077 (47.2)	5853 (66.7)		10,935 (75.9)
		High (n:37,802)		23,427 (84.0)	24,008 (85.7)	26,752 (95.1)	1261 (20.1)	2548 (42.3)	9767 (48.9)	12,577 (71.4)		25,072 (78.9)
		*p* value		< 0.001	< 0.001	< 0.001	0.28	0.083	<0.001	<0.001		<0.001
	**HHI in Men**	Low (n:23,565)		15,134 (81.5)	15,486 (83.0)	17,374 (92.8)	1201 (21.6)	2543 (45.0)	6608 (47.4)			
		Middle (n:14,283)		9302 (83.6)	9532 (85.3)	10,566 (94.1)	574 (19.5)	1664 (44.2)	4192 (50.0)			
		High (n:33,084)		21,571 (85.0)	22,204 (87.1)	24,110 (94.2)	1459 (21.6)	2774(43.6)	9673 (52.0)			
		*p* value		< 0.001	< 0.001	< 0.001	0.033	0.34	< 0.001			
**Not visiting GP**	**HHI in Women**	Low (n:10,775)		4443 (62.2)	4570 (63.7)	5442 (75.5)	107 (13.4)	474 (29.6)	1261 (27.5)	1657 (43.1)		4894 (53.4)
		Middle (n:5663)		2515 (68.1)	2570 (69.2)	3036 (81.2)	55 (16.8)	242 (30.8)	743 (30.8)	1107 (52.7)		3053 (62.6)
		High (n:12,559)		5988 (72.3)	6101 (73.4)	7023 (84.1)	141 (19.1)	383 (34.0)	1612 (31.0)	2856 (59.6)		7764 (68.0)
		*p* value		<0.001	< 0.001	<0.001	0.010	0.050	<0.001	<0.001		<0.001
	**HHI in Men**	Low (n:11,663)		4291 (58.1)	4426 (59.6)	5316 (71.1)	101 (11.4)	394 (27.9)	1239 (25.9)			
		Middle (n:6429)		2603 (63.4)	2696 (65.4)	3213 (77.3)	50 (11.0)	259 (31.9)	741 (28.6)			
		High (n:16,211)		6967 (68.7)	7144 (70.1)	8314 (81.1)	178 (15.2)	476 (32.8)	2022 (32.1)			
		*p* value		< 0.001	< 0.001	< 0.001	0.014	0.013	< 0.001			

*: n (%). GP: General Practitioner. HHI: Household income. BP: blood pressure. Yo: years old. †Measurement in the last 5 year or once a year if you have chronic conditions # Once every year ‡measurement biennial. ¶At least faecal occult blood testing in the last 2 years or once colonoscopy in life ##Once every three years

Among those visiting a GP, women received more BP measurements than men regardless of HHI status. Men with middle and high HHI received more glucose and cholesterol measurements than women, but the proportions were similar among both groups. The highest ratios of cardiometabolic screening and cancer screening benefited those who were more affluent. The greatest HHI differences were found in gynaecologic cancer screening. Women with high HHI were more likely to undergo mammography (71.4% vs. 61.2%, *p* < 0.001) and cervical smear tests (78.9% vs. 69.2%, *p* < 0.001) compared with those with low HHI.

Across the HHI terciles of those who did not attend the GP in the last year, low HHI individuals received fewer preventive services than high HHI subjects. Women who did not attend a GP received more preventive care services than men.

Table 3 displays the association among preventive care services, demographic characteristics, lifestyle, self-perceived health, comorbidities and visits to the GP adjusted by age in a multilevel analysis. The highest odds of receiving preventive care services were observed in individuals who visited the GP in the last year (cardiometabolic screening: adjusted OR: 7.78, 95% CI: 7.43–8.15; colorectal screening adjusted OR: 1.87, 95% CI: 1.80–1.95; mammography adjusted OR: 1.76 2.28, 95% CI: 1.69–1.83 and Pap smear test: adjusted OR: 1.89, 95% CI:1.85–1.94). We also found an association among cardiometabolic screening all preventive care services and having a higher HHI and being a woman. Having bad self-perceived health was related to less breast cancer gynaecological screening but to more of the other preventive care services.

**Table 3:** Multilevel models with associations of GP visits with preventive care services (adjusted OR and 95% confidence interval for covariates)

	Cardiometabolic screening aOR (95% CI) *n*: 162,878	Influenza vaccination in individuals under 65 yo with comorbidities aOR (95% CI) *n*: 39,946	Influenza vaccination in individuals over 65 yo aOR (95% CI) *n*: 35,784	Colorectal screening aOR (95% CI) *n*: 111,984	Mammography aOR (95% CI) *n*: 94,364	Pap smear test aOR (95% CI) *n*:175,623
**Sex** **(reference male)**	1.17 (1.12–1.22)	0.95 (0.90–0.99)	0.98 (0.92–1.03)	0.89 (0.87–0.92)		
**Use of health care services**						
GP visit*	7.78 (7.43–8.15)	1.95 (1.81–2.10)	1.49 (1.34–1.65)	1.87 (1.80–1.95)	1.76 (1.69–1.83)	1.89 (1.85–1.94)
**Household income** **(reference Low)**						
Middle	1.31 (1.24–1.38)	1.08 (1.01–1.15)	1.00 (0.92–1.08)	1.11 (1.06–1.15)	1.05 (1.01–1.09)	1.05 (1.02–1.08)
High	1.57 (1.50–1.65)	1.10 (1.04–1.16)	1.15 (1.08–1.23)	1.22 (1.18–1.26)	0.97 (0.94–1.01)	0.98 (0.95-1.00)
**Lifestyle factors**						
Healthy diet	1.24 (1.19–1.30)	1.18 (1.12–1.25)	1.26 (1.18–1.34)	1.10 (1.06–1.13)	1.81 (1.75–1.87)	1.05 (1.02–1.08)
Tobacco	0.76 (0.73–0.80)	0.68 (0.63–0.73)	0.78 (0.73–0.84)	0.72 (0.70–0.75)	0.68 (0.66–0.71)	0.68 (0.67–0.70)
**Self-perceived health (reference very good and good)**						
Fair	1.19 (1.13–1.26)	1.32 (1.24–1.39)	1.40 (1.31–1.50)	1.47(1.42–1.52)	1.15 (1.12–1.19)	1.07 (1.04–1.10)
Bad and very bad	1.55 (1.41–1.71)	1.38 (1.27–1.51)	1.63 (1.48–1.78)	1.84 (1.75–1.94)	1.13 (1.07–1.19)	0.99 (0.94 − 0.83)
**Comorbidities**						
Obesity	1.03 (0.98–1.09)	0.98 (0.92–1.03)	0.99 (0.93–1.05)	0.89 (0.86–0.92)	0.95 (0.92–0.97)	0.81 (0.79–0.86)
Diabetes	1.38 (1.25–1.32)	1.55 (1.45–1.65)	2.12 (1.97–2.28)	1.01 (0.97–1.06)	0.71 (0.67–0.74)	0.69 (0.66–0.73)
Hypertension	0.43 (0.41–0.45)	1.24 (1.18–1.31)	1.12 (1.03–1.21)	1.07 (1.04–1.10)	0.95 (0.92–0.98)	0.78 (0.75–0.80)
Coronary heart disease	1.05 (0.94–1.17)	1.31 (1.21–1.43)	1.42 (1.29–1.55)	1.18 (1.12–1.25)	0.62 (0.58–0.66)	0.58 (0.54–0.63)
Stroke	1.23 (1.03–1.48)	1.10 (0.95–1.27)	1.19 (1.01-0.39)	0.94 (0.85–1.04)	0.74 (0.65–0.83)	0.65 (0.57–0.75)
Renal disease	1.84 (1.65–2.04)	1.19 (1.07–1.34)	1.02 (0.92–1.14)	1.50 (1.40–1.61)	1.12 (1.03–1.21)	1.13 (1.05–1.21)
**Empty model**						
**ICC**	0.07	0.47	0.41	0.18	0.09	0.03
**MOR**	1.63	5.27	4.25	2.25	1.772	1.40
**Adjusted model**						
**ICC**	0.08	0.53	0.41	0.18	0.09	0.03
**MOR**	1.70	6.36	4.30	2.26	1.72	1.36

aOR (95% CI) = Adjusted odds ratio by age and sex (95% confidence interval). n: describes the target population for each preventive services. Yo: Years old. ICC: intraclass correlation coefficient. MOR: median OR

The role of the country in the likelihood of receiving any of the preventive activities has been calculated through the MOR, showing that an individual from the country with the highest screening has a greater chance of receiving preventive activities compared to a country with lower screening. These differences are more pronounced in influenza vaccination, with an MOR of 6.36 (under 65 years with comorbidities) and 4.30 (over 65 years with comorbidities), followed by colorectal cancer screening, where a patient is 2.26 times more likely to receive screening compared to the country with lower screening, indicating intrinsic country-specific factors related to screening.

## Discussion

### Summary

Cardiometabolic screening was the most commonly utilized preventive care service, followed by colorectal cancer screening and influenza vaccination in individuals over 65 years old. Our study indicates a significant association between visiting a GP in the last year and adherence to all preventive care services. The coverage of all preventive care services improved with increasing HHI, except for influenza vaccination in men. Gender was found to be a factor, with women more likely to undergo cardiometabolic screening but less likely to receive influenza vaccination and colorectal screening compared to men. There is a variability by countries that is very pronounced in the case of influenza and colorectal cancer screening.

### Comparison with literature

Health promotion and disease prevention are two basic roles noted in the definition of primary care. Cardiovascular prevention programs in GP practices have shown a reduction in cardiovascular mortality [27, 28] and are cost-effective [29]. Since the Cochrane review on general health checks in 2019, health checks have been in the spotlight because no impact on morbidity or mortality was found according to the authors [30]. However, there are certain limitations to extrapolating these findings to primary care. The majority of the studies were conducted outside of primary care settings, predominantly in Scandinavian countries, the United Kingdom, and the United States. The cardiovascular risk and cancer prevalence observed in these populations may differ from those in other regions, particularly in the Mediterranean area. Furthermore, these results focused solely on clinical trials, it did not account for opportunistic strategies commonly employed in routine practice. In our study, we scrutinized preventive care services adherence based on guidelines within the outpatient setting across 29 European countries. To comprehensively assess the value of health checks in Europe, further studies involving diverse countries are imperative. As we have described, the variability among countries in the coverage of cardiometabolic and gynaecological screenings (mammography, Pap smear tests) has been lower compared to access to influenza vaccination and colorectal cancer screening. This is due to the organization of screening programs, but also to the population’s acceptance of such screenings. Increased economic investment to expand colorectal cancer screening and influenza vaccination among high-risk groups could promote higher population coverage and reduce disparities in screening access among European individuals.

Although, there are different systems to access primary care (gatekeeper, private insurance, mixed system) [31] and different payments in primary care (fixed salary, pay per service, pay per capita) [32] in Europe, over 70% of patients visited their GPs in the last year in our study, and this pattern was similar to that noted in other European studies [13, 32, 33]. A systematic review [9] found inequalities across socioeconomic statuses in visiting primary care physicians. In our study, minimal differences were observed among individuals who visited the GP across different socioeconomic statuses (low HHI: 36.3% vs. high HHI: 43.3%). There was a significant association between GP visits and adherence to the analysed preventive care services, consistent with findings in the literature regarding influenza vaccination [11, 13], colorectal cancer screening [11, 34], Pap smear testing [35] and mammography [11, 35, 36]. These findings could be explained by the notion that prevention in primary care is delivered not only through a health check-up [36] but also through opportunistic attendance [37–39]. In cardiometabolic screening, our findings surpassed the results reported in other studies (greater than 79.0% in the three services: BP, lipids, glycaemia), whereas other opportunistic screening studies had participation rates between 6 and 64% [40, 41]. This finding could be explained by how prioritization in practice is implemented by GPs [42, 43]. Sebo et al. described that cardiometabolic screening was prioritized over cancer screening by GPs in France and Switzerland [44]. On the other hand, European GPs have a positive attitude towards cardiometabolic screening, especially through an opportunistic approach [45]. The evidence for cardiovascular prevention [27–29] has led to the promotion of guidelines in health promotion in some countries (Supplementary file 2), which could explain the high compliance for cardiovascular screening in primary care [46–48].

Differences in the receipt of preventive care based on sex were not clinically relevant; however, women were more prone to undergo cardiometabolic screening than men (OR: 1.17 (1.12–1.22), as previously described [46, 49]. Another factor associated with compliance with preventive care services was household income among those who visited a GP in the last year. Disparities based on HHI were observed in both sexes, with individuals with low HHI receiving fewer preventive care services compared to those with middle or high HHI, except for influenza vaccination in males. This finding underscores the significance of considering the impact of social determinants on preventive care services and the optimal management of chronic conditions as the complexity of these determinants increases [50]. Similar findings have been described for cardiometabolic screening [51, 52] as well as influenza vaccination and gynaecological screening in women [35, 53]. Our study highlights the importance of visiting a GP to improve the receipt of preventive care services. However, household income emerged as an independent explanatory factor that warrants consideration in this context. Several considerations should be noted. Particularly, addressing inequalities is not a straightforward task; factors such as poverty, housing insecurity, limited social support, and residing in neighbourhoods lacking adequate services can pose significant barriers to accessing the healthcare system [51]. Countries with medical care co-payments could also delay access to some services in low HHI subjects [54]. Additionally, individuals with low HHI mostly/usually have poorer health care experiences, including difficulty in access as well as delays in health care delivery [55] and shorter consultations [56]. The estimated annual time required to provide preventive services ranged from 9.7 to 26.4 min per year in the primary care setting [57, 58], so shorter consultations could make it difficult to receive preventive services as an opportunistic screening. It is important to analyse these barriers and develop methods to improve the receipt of these services in primary care by prioritizing low-HHI subjects and vulnerable populations. Designing community-based cardiometabolic preventive programs and population-based cancer screening programs that are linked to primary care focusing on the low-income group could reduce these inequalities [59–64].

### Strengths and limitations

This study provides a general perspective of the delivery of preventive care services across Europe in primary care. Detailed information by sex and HHI was shared to understand how preventive care was provided in a primary care setting and the role of GPs in these services. Several limitations must be considered. First, the information on cancer screenings includes tests for diagnostic reasons and not only screening programs. Second, the survey did not collect information about health promotion activities in primary care, and this information could be vital to understand the role of GPs in our results. All the answers were self-reported, which could lead to social desirability biases. The number of missing values differs among variables in some member States. We collected the cardiometabolic screening information from the main international organizations, but we could not record information specific to all of the countries as not all the countries had information in English. Other variables by country were not included in the analysis as they were not available in the EHIS data. Additionally, we do not have access to data on the practices, such as panel size, the practice volume or visit length, for inclusion in the analysis; these data could provide a more detailed view of the impact of GPs on the services. Information about the roles of primary care staff is not available in the survey. We believe administrative staff and nurses play an enormous role in prevention, as they have worked with the same population solving doubts about these topics for years. Thus, it would be desirable to collect public statistics about their work in prevention. As we believe prevention is a wider field, all health care professionals should be included in preventive care services research.

### Implications for research and practice

The impact of visiting GPs on compliance with preventive care services and the inequalities in those with low HHI should be highlighted in public policy in Europe. The initiation of new programs, such as The European Cancer Inequalities Registry (ECIR), marks the initial stride toward enhancing the care provided to citizens with low HHI and education [65]. Considerable efforts should be made to harmonise the current guidelines and implement them in primary care offices. This is an essential task to improve the quality of preventive care and specifically to reduce the burden of chronic diseases and medical costs. Furthermore, primary care physicians must have sufficient time to engage their patients in preventive services, especially in the most deprived areas. Our results supported the directives of the WHO regional office for Europe [64] that challenged the Member States to strengthen primary care as the provider for health promotion and disease prevention, among other tasks.

## Conclusions

Visiting the GP in the last year was related to increased compliance with all preventive care services (cardiometabolic screening, cancer screening and influenza vaccination) in Europe. Among those who visited a GP in the last year, more affluent individuals had the highest ratios for cardiometabolic screening and cancer screening. Patterns of compliance among the sexes were found. Women underwent more BP measurements, and men received more influenza vaccinations. Inequalities in access to preventive care services in those with lower household income should lead to an important effort to reduce these inequalities. There is variability in screening performance across countries, with greater disparities observed in influenza vaccination and colorectal cancer screening.

### Electronic supplementary material

Below is the link to the electronic supplementary material.

Supplementary Material 1

## Data Availability

The data that support the findings of this study are available from Eurostat (Eurostat is the statistical office of the European Union) but restrictions apply to the availability of these data under the EU legislation, which were used under license for the current study, and so are not publicly available from the authors but they are available from Eurostat on reasonable request if researchers belong to institutions who have been recognised as research entities by Eurostat [67]. For more information, please contact: ESTAT-Microdata-access@ec.europa.eu

## Declarations

- Directive 95/46/EC of the European Parliament and of the Council of 24 October 1995 on the protection of individuals with regard to the processing of personal data.

- and on the free movement of such data.

- Regulation (EC) No 45/2001 of the European Parliament and of the Council of 18 December 2000 on the protection of individuals with regard to the processing of personal data by the Community institutions and bodies and on the free movement of such data.

- Regulation (EC) No 223/2009 of the European Parliament and of the Council of 11 March 2009 on European statistics and repealing Regulation (EC, Euratom) No 1101/2008 of the European Parliament and of the Council on the transmission of data subject to statistical confidentiality to the Statistical Office of the European Communities, Council Regulation (EC) No 322/97 on Community Statistics, and Council Decision 89/382/EEC, Euratom establishing a Committee on the Statistical Programmes of the European Communities.

- Regulation (EC) No 1338/2008 of the European Parliament and of the Council of 16 December 2008 on Community statistics on public health and health and safety at work.

- Commission Regulation (EU) No 141/2013 of 19 February 2013 implementing Regulation (EC) No 1338/2008 of the European Parliament and of the Council on Community statistics on public health and health and safety at work, as regards statistics based on the European Health Interview Survey (EHIS).

- Regulation (EU) No 557/2013 on access to confidential data for scientific purposes.

Rey Juan Carlos University at Madrid authorized the application of EHIS data for health research purposes, and data were provided by Eurostat upon university request. Eurostat provided anonymized data. Categories with fewer than 49 individuals were not reported to avoid possible identification following the EHIS regulation. This work was additionally approved by the Ethics in Research Committee of the Doce de Octubre Hospital at Madrid city (23/045).

## Abbreviations

BP: Blood pressure
EU: European Union
FOBT: Faecal occult blood testing
GP: General practitioners
HHI: Household income
NCDs: Non-communicable diseases
OR: Odd ratio
WHO: World Health Organization
