# Supplementary material for: Effect of GP visits in the compliance of preventive services: a cross-sectional study in Europe
Source: BMC Prim Care. 2024 May 15;25:165. doi: 10.1186/s12875-024-02400-w (PMC11094967; doi:10.1186/s12875-024-02400-w)
Supplement: Supplementary file 1 — Supplementary Material 1 [file 12875_2024_2400_MOESM1_ESM.docx]

**Supplementary file 1:** Cancer screening according to the latest national recommendations and guidelines (data was collected from official national websites for professionals and patients across Europe. The information was reviewed on 2021)

|  | Colorectal cancer Screening | | | Mammography | | Cervical smear test | |
| --- | --- | --- | --- | --- | --- | --- | --- |
| Country | **Target Population** | **FOBT Frequency** | **Alternative: Colonoscopy, Frequency** | **Target Population** | **Frequency** | **Target Population** | **Frequency** |
| Austria | No universal screening | | | 45-69  40-44 and >70 | Biennial  Voluntary screening | >18 | Annually* |
| Belgium | 50-74 | Biennial |  | 50-69 | Biennial | 25-64 | 3 years |
| Bulgaria | No universal screening | | | 50-69 | No universal screening | 25-64 | No universal screening |
| Croatia | 50-74 | Biennial |  | 50-69 | Biennial | 25-64 | 3 years |
| Cyprus | 50-69 | No universal screening*** | | 50-69 | Biennial | 24-65 | 2 years |
| Czech Republic | 50-54  >55 | Annually  Biennial |  | **≥**45 | Biennial | >15 | Annually* |
| Denmark | 50-74 | Biennial |  | 50-69 | Biennial | 23-64 | 23-49: 3 years  50-64: 5 years |
| Estonia | 60-69 | No universal screening*** | | 50-65 | Biennial | 30-55 | 5 years |
| Finland | 60-68** | Biennial |  | 50-69 | Biennial | 30-60 | 5 years |
| France | 50-74 | Biennial |  | 50-74 | Biennial | 25-65 | 3 years |
| Germany | 50-74 | 50-54 annually  >54: Biennial or Colonoscopy every 10 years | Women can choose colonoscopy if they are ≥55 years  Men can choose between colonoscopy if they are ≥50 years. | 50-69 | Biennial | 20- 60 | 20-35: Annually  ≥35: every 3 years |
| Greece | No universal screening | | | 49-50** | Biennial | 20-69 | Annually |
| Hungary | 50-70 | Biennial |  | 45-65 | Biennial | 25-65 | 3 years |
| Iceland | No universal screening | | | 40-69 | Biennial | 23-65 | 3 years |
| Ireland | 60-69 | Biennial |  | 50-69 | Biennial | 25-65 | 25-29 years: every 3 years  30-65 years: every 5 years |
| Italy | 50-70 | Biennial |  | 50-69  Tuscany region. 45-74 | Biennial | 25-64 | 3 years |
| Latvia | No universal screening | | | 50-69 | Biennial | 25-70 | 3 years |
| Lithuania | 50-74 | Biennial |  | 50-69 | Biennial | 25-60 | 3 years |
| Luxembourg | 55-74 | Biennial |  | 50-69 | Biennial | >15 | Annually* |
| Malta | 56-70 | Biennial |  | 50-69 | 3 years | 27-39** | 3 years |
| Norway | 50-74 | No universal screening*** | | 50-69 | Biennial | 25-69 | 3 years |
| Poland | 55-64 |  | Once in life | 50-69 | Biennial | 25-59 | 3 years |
| Portugal | No universal screening | | | 50-69 | Biennial | 25-64 | 3 years |
| Romania | No universal screening | | | No universal screening | | 25-64 | 5 years |
| Slovakia | 50-75 | Biennial | Patients can choose colonoscopy instead of FOBT | 40-69 | Biennial | 23-64 | 3 years |
| Slovenia | 50-74 | Biennial |  | 50-69 | Biennial | 20-64 | 3 years |
| Spain | 50-69 | Biennial |  | 50-69 | Biennial | 25-65 | 3 years |
| Sweden | 60-69 | No universal screening*** | | 40-74 | Biennial | 23-64 | 23-50: 3 years  51-64: every 7 years |
| The Netherlands | 55-75 | Biennial |  | 50-75 | Biennial | 30-60 | 5 years |
| United Kingdom | 60-74 | Biennial |  | 50-71 | Every 3 years | 25-64 | - 25-49 every 3 years  - 50 to 64 every 5 years |
| Organizations |  | | | | | | |
| US Preventive Services Task Force | 50-75 | Annual | Once every 10 years in case colonoscopy was performed instead of FOBT | 50-75 | Biennial | 21-65 | Every 3 years |
| Canadian Task Force on Preventive Health Care | 50-74 | Biennial | Sigmoidoscopy every 10 years. | 50-74 | Every 2-3 years | 25-69 | Every 3 years |
| EU Cancer Screening | 50-74 |  |  | 50-69 |  | Starting at 20-30 |  |
| Spanish Preventive Services (PAPPS) | 50-74 | Annual or Biennial | Once every 15 years in case colonoscopy was performed instead of FOBT | 50-69 | Biennial | 25-65 | Every 3 years |

* In the course of preventive health check-ups, general practitioners or gynaecologist can make a prescription for a smear test annually.

**Screening programmes have recently started; they have been launched to begin in a target group to be continued as patients get older.

***No universal screening but pilot programmes or regional screenings are taking place.

**Supplementary file 2:** The RECORD statement – checklist of items, extended from the STROBE statement, that should be reported in observational studies using routinely collected health data.

|  | **Item No.** | **STROBE items** | **Location in manuscript where items are reported** | **RECORD items** | **Location in manuscript where items are reported** |
| --- | --- | --- | --- | --- | --- |
| **Title and abstract** | | | | | |
|  | 1 | (a) Indicate the study’s design with a commonly used term in the title or the abstract (b) Provide in the abstract an informative and balanced summary of what was done and what was found |  | RECORD 1.1: The type of data used should be specified in the title or abstract. When possible, the name of the databases used should be included.  RECORD 1.2: If applicable, the geographic region and timeframe within which the study took place should be reported in the title or abstract.  RECORD 1.3: If linkage between databases was conducted for the study, this should be clearly stated in the title or abstract. | 1 |
| **Introduction** | | | | | |
| Background rationale | 2 | Explain the scientific background and rationale for the investigation being reported |  |  | 4-5 |
| Objectives | 3 | State specific objectives, including any prespecified hypotheses |  |  | 5 |
| **Methods** | | | | | |
| Study Design | 4 | Present key elements of study design early in the paper |  |  | 5 |
| Setting | 5 | Describe the setting, locations, and relevant dates, including periods of recruitment, exposure, follow-up, and data collection |  |  | 5 |
| Participants | 6 | *(a) Cohort study* - Give the eligibility criteria, and the sources and methods of selection of participants. Describe methods of follow-up  *Case-control study* - Give the eligibility criteria, and the sources and methods of case ascertainment and control selection. Give the rationale for the choice of cases and controls  *Cross-sectional study* - Give the eligibility criteria, and the sources and methods of selection of participants  *(b) Cohort study* - For matched studies, give matching criteria and number of exposed and unexposed  *Case-control study* - For matched studies, give matching criteria and the number of controls per case |  | RECORD 6.1: The methods of study population selection (such as codes or algorithms used to identify subjects) should be listed in detail. If this is not possible, an explanation should be provided.  RECORD 6.2: Any validation studies of the codes or algorithms used to select the population should be referenced. If validation was conducted for this study and not published elsewhere, detailed methods and results should be provided.  RECORD 6.3: If the study involved linkage of databases, consider use of a flow diagram or other graphical display to demonstrate the data linkage process, including the number of individuals with linked data at each stage. | 5-6 |
| Variables | 7 | Clearly define all outcomes, exposures, predictors, potential confounders, and effect modifiers. Give diagnostic criteria, if applicable. |  | RECORD 7.1: A complete list of codes and algorithms used to classify exposures, outcomes, confounders, and effect modifiers should be provided. If these cannot be reported, an explanation should be provided. | 6-7 |
| Data sources/ measurement | 8 | For each variable of interest, give sources of data and details of methods of assessment (measurement).  Describe comparability of assessment methods if there is more than one group |  |  | 6-7 |
| Bias | 9 | Describe any efforts to address potential sources of bias |  |  | 5-7 |
| Study size | 10 | Explain how the study size was arrived at |  |  | 5-7 |
| Quantitative variables | 11 | Explain how quantitative variables were handled in the analyses. If applicable, describe which groupings were chosen, and why |  |  | 5-8 |
| Statistical methods | 12 | (a) Describe all statistical methods, including those used to control for confounding  (b) Describe any methods used to examine subgroups and interactions  (c) Explain how missing data were addressed  (d) *Cohort study* - If applicable, explain how loss to follow-up was addressed  *Case-control study* - If applicable, explain how matching of cases and controls was addressed  *Cross-sectional study* - If applicable, describe analytical methods taking account of sampling strategy  (e) Describe any sensitivity analyses |  |  |  |
| Data access and cleaning methods |  | .. |  | RECORD 12.1: Authors should describe the extent to which the investigators had access to the database population used to create the study population.  RECORD 12.2: Authors should provide information on the data cleaning methods used in the study. | 15-16 |
| Linkage |  | .. |  | RECORD 12.3: State whether the study included person-level, institutional-level, or other data linkage across two or more databases. The methods of linkage and methods of linkage quality evaluation should be provided. |  |
| **Results** | | | | | |
| Participants | 13 | (a) Report the numbers of individuals at each stage of the study (*e.g.*, numbers potentially eligible, examined for eligibility, confirmed eligible, included in the study, completing follow-up, and analysed)  (b) Give reasons for non-participation at each stage.  (c) Consider use of a flow diagram |  | RECORD 13.1: Describe in detail the selection of the persons included in the study (*i.e.,* study population selection) including filtering based on data quality, data availability and linkage. The selection of included persons can be described in the text and/or by means of the study flow diagram. | 5-6 |
| Descriptive data | 14 | (a) Give characteristics of study participants (*e.g.*, demographic, clinical, social) and information on exposures and potential confounders  (b) Indicate the number of participants with missing data for each variable of interest  (c) *Cohort study* - summarise follow-up time (*e.g.*, average and total amount) |  |  | 8 |
| Outcome data | 15 | *Cohort study* - Report numbers of outcome events or summary measures over time  *Case-control study* - Report numbers in each exposure category, or summary measures of exposure  *Cross-sectional study* - Report numbers of outcome events or summary measures |  |  | 8-10 |
| Main results | 16 | (a) Give unadjusted estimates and, if applicable, confounder-adjusted estimates and their precision (e.g., 95% confidence interval). Make clear which confounders were adjusted for and why they were included  (b) Report category boundaries when continuous variables were categorized  (c) If relevant, consider translating estimates of relative risk into absolute risk for a meaningful time period |  |  | 8-10 |
| Other analyses | 17 | Report other analyses done—e.g., analyses of subgroups and interactions, and sensitivity analyses |  |  | 8-10 |
| **Discussion** | | | | | |
| Key results | 18 | Summarise key results with reference to study objectives |  |  | 10 |
| Limitations | 19 | Discuss limitations of the study, taking into account sources of potential bias or imprecision. Discuss both direction and magnitude of any potential bias |  | RECORD 19.1: Discuss the implications of using data that were not created or collected to answer the specific research question(s). Include discussion of misclassification bias, unmeasured confounding, missing data, and changing eligibility over time, as they pertain to the study being reported. | 13-14 |
| Interpretation | 20 | Give a cautious overall interpretation of results considering objectives, limitations, multiplicity of analyses, results from similar studies, and other relevant evidence |  |  | 10-14 |
| Generalisability | 21 | Discuss the generalisability (external validity) of the study results |  |  | 14 |
| **Other Information** | | | | | |
| Funding | 22 | Give the source of funding and the role of the funders for the present study and, if applicable, for the original study on which the present article is based |  |  | 17 |
| Accessibility of protocol, raw data, and programming code |  | .. |  | RECORD 22.1: Authors should provide information on how to access any supplemental information such as the study protocol, raw data, or programming code. | 17 |

*Reference: Benchimol EI, Smeeth L, Guttmann A, Harron K, Moher D, Petersen I, Sørensen HT, von Elm E, Langan SM, the RECORD Working Committee. The REporting of studies Conducted using Observational Routinely-collected health Data (RECORD) Statement. *PLoS Medicine* 2015; in press.

*Checklist is protected under Creative Commons Attribution ([CC BY](http://creativecommons.org/licenses/by/4.0/)) license.

**Supplementary file 3:** Cardiometabolic screening frequency and target population according to the latest guidelines (reviewed on 2022).

|  | Glucose measurement | | Cholesterol measurement | | BP measurement | |
| --- | --- | --- | --- | --- | --- | --- |
|  | **Target populations**  **(years)** | **Frequency** | **Target populations**  **(years)** | **Frequency** | **Target populations**  **(years)** | **Frequency** |
| Organizations |  |  |  |  |  |  |
| US preventive Services Task Force | 40-70 years who are obese | 3 years | 40-75 | Universal linked to cardiovascular risk score calculation | 18-39  ≥ 40  Obese | 3-5 years  Annual  Annual |
| Canadian Task Force on Preventive Health Care | Not routinely screening if low risk |  | No recent recommendation | | ≥18 | At all appropriate primary care visits |
| NICE | 40-74 | 5 years | ≥ 40 | Universal linked to cardiovascular risk score calculation | ≥ 40 | Every 5 years |
| Spanish Preventive Services (PAPPS) | Not described | Every 4 years | ≥ 18 | Every 4 years | ≥ 40 | Every 3-5 years |
| European Society of Cardiology (ESC) |  |  | Men >40  Women >50 | Every 5 years linked to cardiovascular risk score calculation | Men >40  Women >50 | Every 5 years linked to cardiovascular risk score calculation |
| Comorbidities Follow-up | **Blood test frequency** | | | | **BP measurement** | |
| Dyslipidaemia ESC | Annually | | | | The frequency is not described but it should be Linked to cardiovascular risk score calculation | |
| Hypertension ESC | At least every 2 years | | | | Every 3-6 months | |
| Ischaemic Heart Disease ESC | Every 2 years | | | | They do not describe the frequency | |
| Stroke, Royal College of Physicians | At least 3 months later than the stroke episode  (They don´t describe the further follow-up) | | | | It should be monitored frequently | |
| Diabetes, ADA | At least two times a year | | | | In every routine clinical visit. | |
| Renal Disease, KDIGO | More frequently than annually | | | | They do not describe the frequency | |

ESC: European Society of Cardiology. ADA: American Association of Diabetes. KDIGO: Kidney Disease Improving Global Outcomes

**Supplementary file 4**: Population characteristics according to sex and visiting or not their GP in the last year

| Women (n=129967) | | | | Men (n=112245) | | |
| --- | --- | --- | --- | --- | --- | --- |
|  | **GP visit in the last year** | **Not visiting GP in the last year** | p value | **GP visit in the**  **last year** | **Not visiting GP in the last year** | p value |
| n | 99257 (76.3) | 30710 (23.6) |  | 75790 (67.5) | 36455 (32.4) |  |
| Urban* | 65571 (66.1) | 20329 (66.3) | 0.66 | 49051 (64.8) | 23535 (64.6) | 0.60 |
| Age* 25-39 years | 23751 (23.9) | 9785 (31.9) | <0.001 | 16687 (22.0) | 12671 (34.8) | <0.001 |
| 40-64 years | 54649 (55.1) | 17171 (55.9) |  | 42321 (55.8) | 19941 (54.7) |  |
| 65-74 years | 20857 (21.0) | 3754 (12.2) |  | 16782 (22.1) | 3843 (10.5) |  |
| Household income* |  |  |  |  |  |  |
| Low | 35825 (38.7) | 10775 (37.2) |  | 23565 (33.2) | 11663 (34.0) | <0.001 |
| Middle | 18968 (20.5) | 5663 (19.5) |  | 14283 (20.1) | 6429 (18.7) |  |
| High | 37802 (40.8) | 12559 (43.3) |  | 33084 (46.6) | 16211 (47.3) |  |
| Self-perceived health* |  |  |  |  |  |  |
| Very good and good | 60356 (62.0) | 25268 (84.6) | <0.001 | 47271 (64.4) | 29769 (85.3) | <0.001 |
| Fair | 27753 (28.5) | 3947 (13.2) |  | 19416 (26.4) | 4399 (12.6) |  |
| Bad and very bad | 9234 (9.5) | 644 (2.2) |  | 6766 (9.2) | 751 (2.2) |  |
| Lifestyle Factors* |  |  |  |  |  |  |
| Healthy diet | 69960 (70.5) | 20915 (68.1) | <0.001 | 43952 (58.0) | 19220 (52.7) | <0.001 |
| Exercise | 97207 (97.9) | 30125 (98.1) | 0.081 | 74055 (97.7) | 35740 (98.0) | 0.001 |
| Tobacco | 20047 (20.5) | 6951 (23.0) | <0.001 | 21083 (28.2) | 13260 (37.1) | <0.001 |
| Alcohol abuse | 179 (0.2) | 54 (0.2) | 0.87 | 3970 (5.5) | 2202 (6.3) | <0.001 |
| Chronic Conditions* |  |  |  |  |  |  |
| Obesity | 21563 (21.7) | 4599 (15.0) | <0.001 | 6473 (17.8) | 17522 (23.1) | <0.001 |
| Diabetes | 7230 (7.4) | 565 (1.9) | <0.001 | 792 (2.2) | 7340 (9.8) | <0.001 |
| Hypertension | 26116 (26.6) | 2666 (8.8) | <0.001 | 21664 (28.9) | 3279 (9.1) | <0.001 |
| Coronary heart disease | 4113 (4.2) | 297 (1.0) | <0.001 | 4776 (6.4) | 504 (1.4) | <0.001 |
| Stroke | 1163 (1.2) | 94 (0.3) | <0.001 | 1305 (1.8) | 148 (0.4) | <0.001 |
| Renal disease | 3234 (3.3) | 284 (0.9) | <0.001 | 2319 (3.1) | 345 (1.0) | <0.001 |

*: n (%). GP: General Practitioner

**Supplementary file 5:** Individual characteristics based on preventive services

|  | All | Cardiometabolic screening | | | Influenza vaccination | | Cancer screening | | |
| --- | --- | --- | --- | --- | --- | --- | --- | --- | --- |
|  |  | **Glucose measurement** | **Cholesterol measurement** | **BP measurement** | **Individuals under 65 years old with comorbidities** | **Individuals over 65 years old** | **Colorectal screening** | **Mammography** | **Pap smear**  **test** |
| Target population | 242212 | 176890 | 177642 | 178427 | 45209 | 45209 | 126276 | 106260 | 196976 |
| N | 242212 | 139863 (79.0) | 143286 (80.6) | 161193 (90.3) | 7522 (40.1) | 18138 (40.1) | 55723 (44.1) | 36417 (34.2) | 75088 (38.1) |
| Women* | 129967 (53.7) | 76162 (54.5) | 77887 (54.4) | 87979 (54.6) | 3802 (50.5) | 9601 (52.9) | 29772 (53.4) | 36417 (100) | 75088 (100) |
| Urban* | 158486 (65.5) | 91216 (65.3) | 93390 (65.2) | 104537 (64.9) | 5309 (70.7) | 12656 (69.9%) | 36896 (66.3) | 24090 (66.2) | 50167 (66.9) |
| Age* |  |  |  |  |  |  |  |  |  |
| 25-39years | 62894 (26.0) | 2432 (1.7) | 2300 (1.6) | 3289 (2.0) | 471 (6.3) |  |  |  | 25072 (33.4) |
| 40-64 years | 134082 (55.4) | 101444 (72.5) | 103932 (72.5) | 117265 (72.7) | 7051 (93.7) |  | 33256 (59.7) | 28449 (78.1) | 50016 (66.6) |
| 65-74 years | 45236 (18.7) | 35987 (25.7) | 37054 (25.9) | 40639 (25.2) |  | 18138 | 22467 (40.3) | 7968 (21.9) |  |
| Household income* |  |  |  |  |  |  |  |  |  |
| Low | 81828 (36.1) | 46496 (35.5) | 47623 (35.5) | 54351 (35.9) | 2872 (39.8) | 7004 (40.7) | 18606 (35.5) | 11579 (34.1) | 23545 (33.5) |
| Middle | 45343 (20.0) | 26482 (20.2) | 27145 (20.2) | 30692 (20.3) | 1306 (18.1) | 4026 (23.4) | 10753 (20.5) | 6960 (20.5) | 13988 (19.9) |
| High | 99656 (43.9) | 57953 (44.3) | 59457 (44.3) | 66199 (43.8) | 3039 (42.1) | 6183 (35.9) | 23074 (44.0) | 15433 (45.4) | 32836 (46.7) |
| Self-perceived health* |  |  |  |  |  |  |  |  |  |
| Very good and good | 162664 (69.1) | 84923 (61.1) | 87622 (61.6) | 99266 (62.0) | 3079 (41.5) | 8530 (49.4) | 29049 (54.9) | 21193 (58.4) | 55730 (74.4) |
| Fair | 55515 (23.6) | 40207 (28.9) | 40842 (28.7) | 45530 (28.4) | 2980 (40.1) | 6471 (37.5) | 17549 (33.2) | 11552 (31.8) | 15263 (20.4) |
| Bad and very bad | 17395 (7.4) | 13806 (9.9) | 13834 (9.7) | 15283 (9.5) | 1364 (18.4) | 2267 (13.1) | 6283 (11.9) | 3552 (9.8) | 3912 (5.2) |
| Lifestyle Factors* |  |  |  |  |  |  |  |  |  |
| Healthy diet | 154047 (63.6) | 94308 (67.4) | 96518 (67.4) | 107324 (66.6) | 4915 (65.3) | 12784 (70.5) | 37589 (67.5) | 27641 (75.9) | 54291 (72.3) |
| Exercise | 237127 (97.9) | 137065 (98.0) | 140426 (98.0) | 157930 (98.0) | 7398 (98.4) | 18006 (99.3) | 54723 (98.2) | 35738 (98.1) | 73127 (97.4) |
| Tobacco | 61341 (25.7) | 31586 (22.8) | 32306 (22.8) | 37005 (23.2) | 1618 (21.8) | 1934 (10.9) | 9709 (17.9) | 6650 (18.4) | 17302 (23.2) |
| Alcohol abuse | 6405 (2.8) | 3728 (2.8) | 3981 (3.0) | 256 (4.0) | 278 (3.9) | 645 (3.8) | 1695 (3.3) | 70 (0.2) | 113 (0.2) |
| Chronic Conditions* |  |  |  |  |  |  |  |  |  |
| Obesity | 50157 (20.7) | 31170 (22.3) | 31599 (22.1) | 2645 (38.5) | 2865 (38.1) | 5132 (28.3) | 14419 (25.9) | 8335 (22.9) | 12174 (16.2) |
| Diabetes | 15927 (6.7) | 14381 (10.3) | 13700 (9.6) | 4484 (66.1) | 2466 (33.3) | 3460 (19.8) | 6625 (12.2) | 3036 (8.5) | 2622 (3.5) |
| Hypertension | 53725 (22.4) | 40151 (28.8) | 40999 (28.7) | 5256 (76.7) | 5744 (76.6) | 8747 (49.3) | 21046 (38.4) | 11842 (32.9) | 11227 (15.1) |
| Coronary heart disease | 9690 (4.1) | 7896 (5.7) | 8084 (5.7) | 920 (13.6) | 999 (13.6) | 1911 (11.0) | 4066 (7.5) | 1538 (4.3) | 1245 (1.7) |
| Stroke | 2710 (1.1) | 2127 (1.5) | 2168 (1.5) | 2398 (1.5) | 286 (3.9) | 537 (3.1) | 1133 (2.1) | 450 (1.3) | 361 (0.5) |
| Renal disease | 6182 (2.6) | 4661 (3.4) | 4661 (3.3) | 5309 (3.3) | 645 (8.8) | 891 (5.1) | 2390 (4.4) | 1233 (3.4) | 1588 (2.1) |

*: n (%). BP: blood pressure Healthy diet: eating vegetables and fruits at least 4-6 days a week. Active life: exercise practice of ≥ 2.5 hours/week. Alcohol abuse: daily consumption over 20g in women and 40 mg in men daily.
